# Microbial Community Succession During Bioremediation of Petroleum-Contaminated Soils Using *Rhodococcus* sp. OS62-1 and *Pseudomonas* sp. P35

**DOI:** 10.3390/microorganisms14010007

**Published:** 2025-12-19

**Authors:** Xiaodong Liu, Yuxi Ma, Yingying Jiang, Yidan Guo, Zhenshan Deng, Xiaolong He

**Affiliations:** 1 Shaanxi Key Laboratory of Research and Utilization of Resource Plants on the Loess Plateau, College of Life Sciences, Yan’an University, Yan’an 716000, China; 15891236204@163.com (Y.M.); jyy@yau.edu.cn (Y.J.); 15129133773@163.com (Y.G.); zhenshandeng214@163.com (Z.D.); ydhelong@163.com (X.H.); 2 Engineering Research Center of Microbial Resources Development and Green Recycling, University of Shaanxi Province, Yan’an University, Yan’an 716000, China

**Keywords:** petroleum-degrading bacteria, biodegradation, *Rhodococcus* sp. OS62-1, *Pseudomonas* sp. P35, collaborative microbial remediation

## Abstract

Oil pollution poses a persistent threat to soil ecosystems globally, and bioremediation using bacterial consortia has emerged as a cost-effective remediation strategy. However, the role of weak petroleum-degrading bacteria in enhancing the efficiency of specialized petroleum-degrading bacteria remains unclear. This study explores the synergy and remediation potential of a two-bacterial consortium: the petroleum-degrading bacterium *Rhodococcus* sp. OS62-1 and the weak petroleum-degrading bacterium *Pseudomonas* sp. P35. A 25-day microcosm experiment was conducted with petroleum-contaminated soil, and four treatments were set: (1) uninoculated control, (2) inoculation with *Rhodococcus* sp. OS62-1 alone, (3) inoculation with *Pseudomonas* sp. P35 alone, and (4) inoculation with the consortium. Soil samples were collected periodically to analyze petroleum degradation efficiency, soil enzyme activities (dehydrogenase, catalase, polyphenol oxidase, and lipase), and microbial community composition (16S rRNA gene sequencing). Inoculating the soils with this consortium produced a higher petroleum degradation rate, microbial activity, and soil enzyme activity than inoculation with strain OS62-1 or P35 alone. Inoculation with strain P35 also contributed to the maintenance of strain OS62-1 during bioremediation. The study of microbial community structure found that the relative abundance of phylum Acidobacteriota (57.6 ± 5.3% to 75.6 ± 8.1%) and the *Nocardioides* genus (36.4 ± 4.5% to 53.0 ± 9.2%) increased dramatically during the bioremediation process. Pearson’s correlation analysis revealed that inoculation with strain OS62-1 and/or strain P35 increases the soil enzyme activity, boosts native oil-degrading bacteria, and accelerates the degradation of petroleum contaminants. Molecular ecological networks analysis revealed that inoculation with strain OS62-1 and/or strain P35 increased the complexity and robustness of the microbial network. These findings confirm that weak petroleum-degrading bacteria can synergistically enhance the bioremediation efficiency of specialized petroleum-degrading bacteria, providing a practical strategy for optimizing the design of bacterial consortia in the bioremediation of oil-polluted soils.

## 1. Introduction

Oil pollution is a serious global environmental problem [1]. Large quantities of oil are spilled into the environment due to both anthropogenic activities and natural causes, which causes serious pollution [2]. Many oil components do not degrade quickly in the environment and accumulate in humans and animals through the food chain, causing serious health problems [3,4]. The remediation of oil pollution typically involves biological, physical, and chemical methods [5]. Physicochemical methods have the disadvantages of high cost, inconvenient operation, and can easily produce secondary pollution, whereas biological methods have the advantages of environmental friendliness and economic simplicity, and thus, have received more attention [6,7]. Bioremediation commonly includes the process of bioaugmentation and biostimulation [8]. Bioaugmentation is the process of introducing pollutant-degrading microorganisms into the soil, while biostimulation enhances pollutant degradation by adding nutrients to promote the activity of indigenous degrading microorganisms [9].

Petroleum-degrading bacteria are the key factors in the bioremediation of oil pollution [10]. Owing to the complexity of crude oil components, the degradation effect of a single strain is often limited. The use of a combination of microorganisms for bioremediation has many advantages, such as a wider range of substrate spectra, increased robustness, and improved adaptation to complex environments [11,12]. *Rhodococcus ruber* SS-4 and *Pseudomonas putida* SW-3 were added to sludge and sewage for enhanced bioremediation and showed improved degradation rates of 97.9% and 92.7%, respectively [13]. The consortium of *Dietzia* sp. CN-3 and *Acinetobacter* sp. HC8-3S not only had a higher oil-degradation efficiency but also had a stronger pH and NaCl tolerance than a single strain [14]. Therefore, the use of microbial flora in environmental remediation is of great significance.

*Rhodococcus* is widely distributed, has strong stress resistance and catabolic abilities, and is considered an ideal candidate for environmental remediation [15,16]. Studies have shown that many *Rhodococcus* species have oil-degrading abilities and play important roles in bioremediation [17,18,19]. *Rhodococcus* sp. OS62-1, with petroleum-degrading ability, and *Pseudomonas* sp. P35, with weak petroleum-degrading ability, were both isolated from enrichment cultures of petroleum-degrading bacteria [20]. The consortium combined with *Rhodococcus* sp. OS62-1 and *Pseudomonas* sp. P35 showed higher petroleum-degrading efficiency than a single strain [20]. Due to the significant difference between the soil environment and the flask environment, the oil degradation of the consortium in the soil still needs further study.

In this study, we aimed to explore the effects and mechanisms of the bioremediation of petroleum-contaminated soil by the consortium (*Rhodococcus* sp. OS62-1 and *Pseudomonas* sp. P35). The objectives were to (1) assess the bioremediation efficiencies of the consortium, (2) investigate the changes in microbial community structures and microbial networks, and (3) analyze the changes in soil enzyme activities.

## 2. Materials and Methods

### 2.1. Experimental Designs

The uncontaminated soil used in the experiment was collected from a mountain behind Yan’an University (109°46′51″ E, 36°6′21″ N). Debris, such as branches and leaves, and other impurities were removed from the soil, and the soil was air-dried for later use. Crude oil was collected from the Yanchang Oilfield in northern Shaanxi, China. An appropriate amount of crude oil was dissolved in petroleum ether and mixed with uncontaminated soil. The petroleum ether was completely volatilized after standing for 5 days, and was used as a follow-up test for artificially simulated crude oil-contaminated soil, designated as BS. The final crude oil concentration in the BS soil was approximately 5000 mg/kg.

*Rhodococcus* sp. OS62-1 (petroleum-degrading bacterium) and *Pseudomonas* sp. P35 (weak petroleum-degrading bacterium) bacterial cells were both isolated from enrichment cultures of petroleum-degrading bacteria. The oil degradation rate of strain OS62-1 can reach 72.28 ± 3.60%, while that of strain P35 is only 13.61 ± 4.12% [20]. The strain OS62-1 and P35 bacterial cells were cultured and prepared as a consortium as described previously [20]. Briefly, the purified strains OS62-1 and P35 were first inoculated into PYG liquid medium (peptone (5 g·L^−1^), yeast extract (0.5 g·L^−1^), glucose (5 g·L^−1^), beef extract (3 g·L^−1^), MgSO_4_·7H_2_O (1.5 g·L^−1^) and NaCl (10 g·L^−1^) at pH 7.5) and cultured at 28 °C and 160 rpm for 3 days. Then, the cells were collected by centrifugation at 8000 rpm, and the pellets were washed 3 times using sterile water. Finally, the cells were resuspended in sterile water and adjusted to OD_600_ = 1.0.

### 2.2. Experiment Design

**Figure 1:**
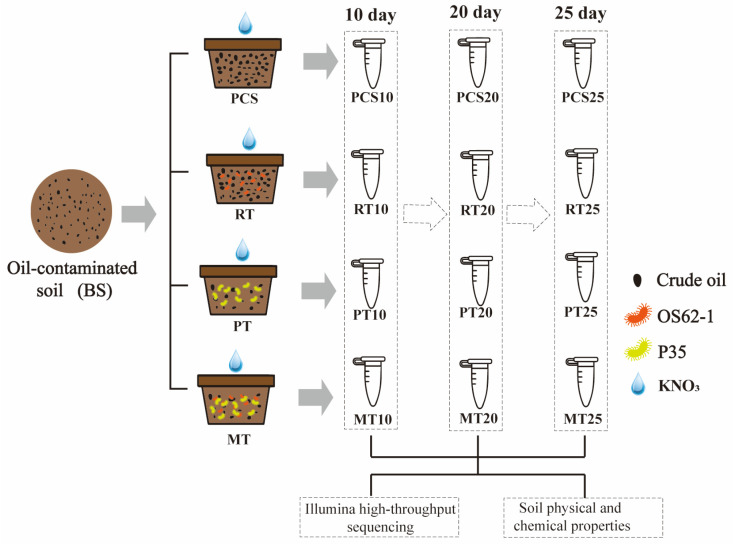
Schematic diagram of bioremediation experimental design and sampling times.

The following four treatments were applied to the BS soil, and 5 pots were used for each treatment (Figure 1):

(1) KNO_3_ solution alone (PCS): 1 kg of BS soil was placed into flowerpots; KNO_3_ solution was added to achieve a final concentration of nitrate of approximately 500 mg/kg.

(2) KNO_3_ solution and *Rhodococcus* sp. OS62-1 (RT): All other experimental conditions were the same as those described above (1), except that *Rhodococcus* sp. OS62-1 was added to each pot. A suspension of *Rhodococcus* sp. OS62-1 was adjusted to OD_600_ = 1.0 (~6.5 × 10^8^ CFU/mL), and 5 mL was added to each pot.

(3) KNO_3_ solution and *Pseudomonas* sp. P35 (PT): All other experimental conditions were the same as those described above (1), except that *Pseudomonas* sp. P35 was added to each pot. A suspension of *Pseudomonas* sp. P35 was adjusted to OD_600_ = 1.0 (~5.7 × 10^8^ CFU/mL), and 5 mL was added to each pot.

(4) KNO_3_ solution and consortium (MT): All other experimental conditions were the same as those described above (1); a combination of *Rhodococcus* sp. OS62-1 and *Pseudomonas* sp. P35 was added to each pot. The suspension of *Rhodococcus* sp. OS62-1 (OD_600_ = 1.0) and *Pseudomonas* sp. P35 (OD_600_ = 1.0) were mixed equally in volume, and 5 mL was added to each pot.

Since a large amount of nitrogen is consumed during the petroleum degradation process in soil, to prevent the interruption of bioremediation due to nitrogen deficiency, a certain amount of potassium nitrate solution was added to all control and experimental groups as nitrogen supplementation.

To raise the moisture content of dry soil to 20%, we first calculated the required water amount based on the soil quality. Subtract the water volume used for adding potassium nitrate solution and bacterial suspension, and then supplement the remaining water to bring the initial soil moisture content to 20%. After adjusting the initial moisture, weigh the pot and record the weight. Subsequently, weigh the pot every 3 days to calculate the water evaporation weight. Add the corresponding amount of sterile water according to the evaporation weight to maintain the soil moisture at around 20%.

Soil samples were collected at three time points (10, 20, and 25 days). At each sampling time, soil (50 g) from each pot was sampled using a five-point method. Subsamples from these five points were then homogenized to form a single composite sample per pot for subsequent analysis. Briefly, 5 g of soil was stored at −80 °C for Illumina MiSeq sequencing, and the remaining 45 g was used for soil physicochemical parameters analysis and enzyme activity tests.

### 2.3. Analysis of Soil Properties

The soil samples were collected, air-dried, and impurities were removed by filtering the soil through a 2 mm sieve. The crude oil concentrations of the treated soil samples were tested as described in Soil—Determination of petroleum oil—Infrared spectrophotometry HJ 1051-2019 [21]. The concentrations of soil nitrate and nitrite were measured as described in Soil-Determination of ammonium, nitrite and nitrate by extraction with potassium chloride solution—spectrophotometric methods HJ 634-2012 [22]. The soil total nitrogen concentration was measured as described in Soil quality-Determination of total nitrogen—Modified Kjeldahl HJ 717-2014 [23]. The soil enzyme activity was measured using fresh soil samples. The soil catalase (CAT, BC0100), dehydrogenase (DHA, BC0390), polyphenol oxidase (PPO, BC0110), and lipase (LPS, BC3980) levels were determined using appropriate kits (Beijing Solarbio Science & Technology Co., Ltd., Beijing, China). Specific assays were performed according to the manufacturer’s instructions.

### 2.4. Microbial Community Analysis

The microbial communities in the original soil (BS) and in the samples collected on days 10, 20, and 25 were investigated. Soil (5 g) from each sample was sent to Biomarker Technologies (Beijing, China) for DNA extraction, library construction, and amplicon sequencing. DNA was extracted using a Fast DNA SPIN Kit (MP Biomedicals, Santa Ana, CA, USA) according to the manufacturer’s protocol. The quality and quantity of DNA were determined using 1% agarose gel electrophoresis and a NanoDrop 2000 spectrophotometer (Thermo Scientific, Waltham, MA, USA). Primers 338F (5′-ACTCCTACGGGAGGCAGCA-3′) and 806R (5′-GGACTACHVGGGTWTCTAAT-3′) were used for 16S rRNA gene (V3-V4 region) sequencing under the following conditions: denaturation at 95 °C for 10 min, followed by 40 cycles of 95 °C for 15 s, 55 °C for 60 s, and 72 °C for 90 s, with a final hold at 72 °C for 7 min. Amplicon sequencing was performed using the Illumina NovaSeq 6000 platform. Raw reads were filtered using Trimmomatic (ver. 0.33) [24], and primers were identified and removed using Cutadapt (ver. 1.9.1) [25]. The amplicon sequence variants (ASVs) were obtained using the QIIME2 dada 2.0 plugin [26,27]. Contaminated mitochondrial and chloroplast sequences were filtered and removed. To minimize the effects of sequencing depth on the data analysis, the number of reads in each sample was reduced to 34,356. The generated ASVs were aligned to the GREENGENES 13_8 database classifier with 99% similarity using the QIIME2 feature-classifier plugin to generate a taxonomic table. The 16S rRNA Illumina libraries were deposited in the NCBI small read archive (SRA) dataset under the SRA accession numbers SRR29243594—SRR29243658.

### 2.5. Analysis of Microbial Network

The molecular ecological networks (MENs) of each soil sample were constructed using R software (4.5.2) [28,29]. Pearson’s coefficients between the ASVs in the sample were calculated using the ‘dplyr’ (ver. 1.1.4) and ‘Hmisc’ (ver. 5.2-4) packages. If *p* < 0.05 and r > 0.4, the two ASVs were considered to be correlated; otherwise, there is no correlation. Subsequently, the ‘igraph’ package was used to color and visualize the network according to the modules. The number of network nodes (n), number of edges (L), edge density (Con), average degree (Average K), relative modulus (RM), and robustness were calculated using the ‘ggCLusterNet’ package (https://github.com/taowenmicro/ggClusterNet, access on 16 December 2025) [30].

### 2.6. Statistical Analysis

Each treatment was conducted five times. The experimental results are expressed as the mean ± standard deviation (SD). The normality was tested using the Kolmogorov–Smirnov test, and the homogeneity of variance was tested using the Levene test (car package). One-way ANOVA analysis was conducted before the Tukey HSD test (MASS package) (*p* < 0.05). R software (4.5.2) and R Studio (2025.09.0) were used for data processing and drawing.

## 3. Results and Discussion

### 3.1. Soil Physicochemical Parameters During Bioremediation

The different bioremediation methods showed different oil degradation efficiencies. After 25 days of repair, the residual oil concentrations in the PCS, PT, RT, and MT groups were 4214.33 ± 122.71 mg/kg (21.07 ± 3.94% removal efficiency), 3508.78 ± 192.59 mg/kg (34.23 ± 5.39% removal efficiency), 2468.79 ± 148.22 mg/kg (53.78 ± 2.93% removal efficiency), and 2271.87 ± 137.63 mg/kg (63.08 ± 2.96% removal efficiency), respectively (Table 1). Application of *Rhodococcus* sp. OS62-1 and *Pseudomonas* sp. P35 (MT group) significantly enhanced the decomposition of crude oil contaminants compared with the application of *Rhodococcus* sp. OS62-1 (RT group) alone and *Pseudomonas* sp. P35 (PT group) alone, indicating that *Pseudomonas* sp. P35 assists *Rhodococcus* sp. OS62-1 in degrading oil contaminants. Some *Pseudomonas* species have good oil degradation ability [2,31,32,33], but previous studies have shown that the oil degradation ability of *Pseudomonas* sp. P35 is weak. In flask tests, the oil degradation rate of *Pseudomonas* sp. P35 in 7 days is only 13.61 ± 4.12% [20]. Some *Pseudomonas* spp. can promote the biodegradation of soil oil by producing surfactants [34,35] or through metabolic exchange [36]. Our results showed that biosurfactant production by *Pseudomonas* sp. P35 was relatively weak (unpublished data). Therefore, we speculated that *Pseudomonas* sp. P35 may promote the oil-degrading ability of *Rhodococcus* sp. OS62-1 in soil through metabolic coupling or stimulation of other degraders.

Nitrogen is a limiting factor in the bioremediation of oil pollution in many cases [37,38,39]. We added KNO_3_ solution to the soil at the beginning of the experiment until the nitrate-nitrogen content reached approximately 500 mg/kg. The results showed that the higher the oil degradation rate, the lower the nitrate nitrogen content, indicating that nitrogen supplementation is essential for crude oil bioremediation [38]. After 25 days, the residual nitrate concentrations in the PCS, PT, RT, and MT groups were 457.52 ± 30.09, 352.82 ± 24.99, 173.39 ± 18.90, and 115.78 ± 15.71 mg/kg, respectively (Table 1). The trend of the change in total nitrogen was consistent with that of nitrate nitrogen. Microorganisms in the soil reduce nitrate nitrogen to nitrite nitrogen via denitrification [40]. Therefore, the nitrite nitrogen content of soil reflects the intensity of microbial denitrification to a certain extent. After 25 days of remediation, the nitrite nitrogen content in the PT, RT, and MT groups was significantly higher than that in the PCS group, indicating that the metabolic activity of microorganisms in the soil was enhanced after the addition of exogenous microorganisms. The change in nitrogen content in the remediation system has an important relationship with soil microbial activity. The decrease in total nitrogen and nitrate nitrogen content and the increase in nitrite nitrogen content indicated enhanced soil microbial activity. Microbial activity is an important factor in the degradation of petroleum pollutants in soil [41,42]. Therefore, the addition of microbial agents resulted in higher crude-oil degradation rates than those in the PCS group without microbial agents. Owing to the weak ability of *Pseudomonas* to degrade petroleum [16], the total nitrogen, nitrate nitrogen, and nitrite nitrogen contents in the PT group were higher than those in the RT and MT groups (Table 1). The MT group exhibited the highest oil-degradation efficiency, nitrate nitrogen consumption, and nitrite nitrogen production.

### 3.2. Soil Enzyme Activities

**Figure 2:**
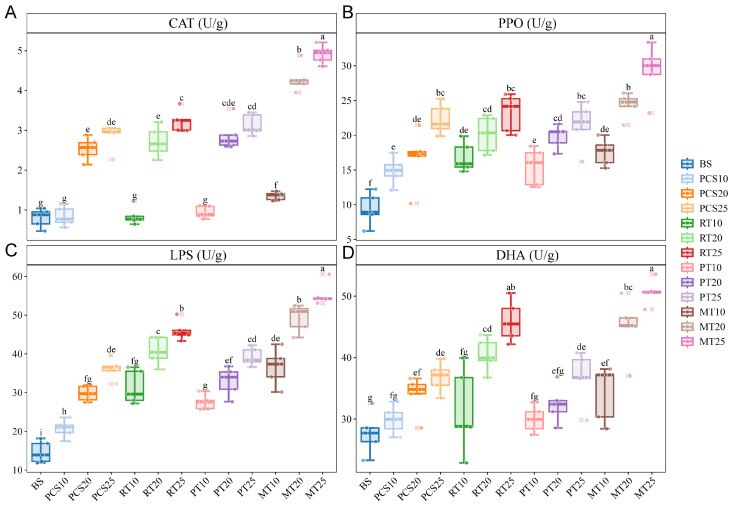
Soil enzyme activities in the BS soil and the four bioremediation experiments at different times: (**A**) catalase (CAT) activity; (**B**) polyphenol oxidase (PPO) activity; (**C**) lipase (LPS) activity; (**D**) dehydrogenase (DHA) activity. Each sample contained five replicates. Different lowercase letters indicate significant differences (*p* < 0.05) based on a one-way ANOVA, followed by a Tukey’s test.

In general, soil enzymes are believed to be derived mainly from soil microbes [43]. Soil enzyme activity reflects the metabolic activity of microorganisms and plays an important role in soil bioremediation [44]. In this study, we evaluated the effects of different bioremediation treatments on the metabolic function of the soil microbes based on the activities of several key enzymes related to petroleum degradation, including CAT, DHA, PPO, and LPS (Figure 2).

CAT promotes the detoxification of H_2_O_2_ produced by microorganisms under environmental stress [45]. The CAT activity increased in all treatments over time (Figure 2A). On day 10, the CAT enzyme activities of the four treatment groups were not significantly different from those of the BS group, except in the MT group, which was significantly higher than in the BS group. A sharp increase was detected from day 10 to 20 in the four treatments, but this increase slowed down from day 20 to 25. There were some differences between the results of the present study and those of [45,46], which both showed a sharp increase in CAT activity from 0 to 8 days. This difference may be due to the different treatments and bacteria used.

PPO is an important enzyme that degrades recalcitrant organic materials into unstable carbon, especially cyclic hydrocarbon ring openings [47,48]. The increase in PPO activity was moderate (Figure 2B). Unlike the CAT activity, the PPO activity significantly increased on day 10 in the four bioremediation treatments compared to the BS group. Similar results were observed for LPS activity (Figure 2C), an important enzyme involved in petroleum hydrocarbon degradation [45]. The trends in PPO and LPS activity were similar to those of previous studies [45,49].

DHA is an oxidoreductase enzyme involved in the degradation of organic compounds [50]. DHA activity on day 10 in the four treatments was not significantly different from that in the BS group, except for the MT group, which was significantly higher than that of the BS group (Figure 2D). The increase in DHA activity was slow in the PCS group, whereas the increases in the RT, PT, and MT groups were sharp, similar to that observed by [46].

For all samples, MT25 possessed the highest enzyme activity except for DHA in RT25 (higher but not significant), indicating that the microbial activity in the MT group was higher than that in the other groups. The CAT activity in the PCS, RT, and PT groups at each time point was similar, which was similar to that observed for PPO activity. Because the RT and PT groups also supplemented with KNO_3_ solution similar to the PCS group, the CAT and PPO activities of the PCS, RT, and PT groups indicated that the KNO_3_ solution had a more significant influence than the inoculated bacteria. However, the MT group exhibited the highest CAT and PPO activities, indicating that *Rhodococcus* sp. OS62-1 and *Pseudomonas* sp. P35 cooperated to promote the soil enzyme activities of CAT and PPO. The RT group showed higher LPS activity than the PCS and PT groups, indicating that supplementation with *Rhodococcus* sp. OS62-1 enhanced the soil LPS activity. Many *Rhodococcus* spp. produce LPS, which may partially explain the higher LPS enzyme activity observed in the RT and MT groups [51,52]. A similar trend was observed for DHA. In summary, supplementation with a KNO_3_ solution enhanced the activities of the four soil enzymes, and supplementation with *Rhodococcus* sp. OS62-1 further enhanced LPS and DHA activities. *Rhodococcus* sp. OS62-1 and *Pseudomonas* sp. P35 can synergistically enhance the activity of the four soil enzymes tested.

### 3.3. Microbial Composition and Diversity

#### 3.3.1. Microbial Alpha Diversity

Changes in the soil environmental conditions can affect soil microbial diversity. The alpha diversity of all treatment groups decreased during the experimental period, and the species diversity indices (Shannon and Simpson indices) of all treatments were significantly lower than those of the BS group. Although the species richness index (Chao1 index) of some samples (RT10, PT10, and MT10) was not significantly different compared to that of the BS group, it was clearly lower (Table 2). During the experimental period, there were no significant differences in microbial species richness or diversity among the groups. These results revealed that oil pollution can reduce soil microbial diversity, which is similar to the findings of [53]. Although bioremediation can decrease the oil content in soils, it cannot restore soil microbial species diversity to that of uncontaminated soil in the short term.

#### 3.3.2. Microbial Community Structure

**Figure 3:**
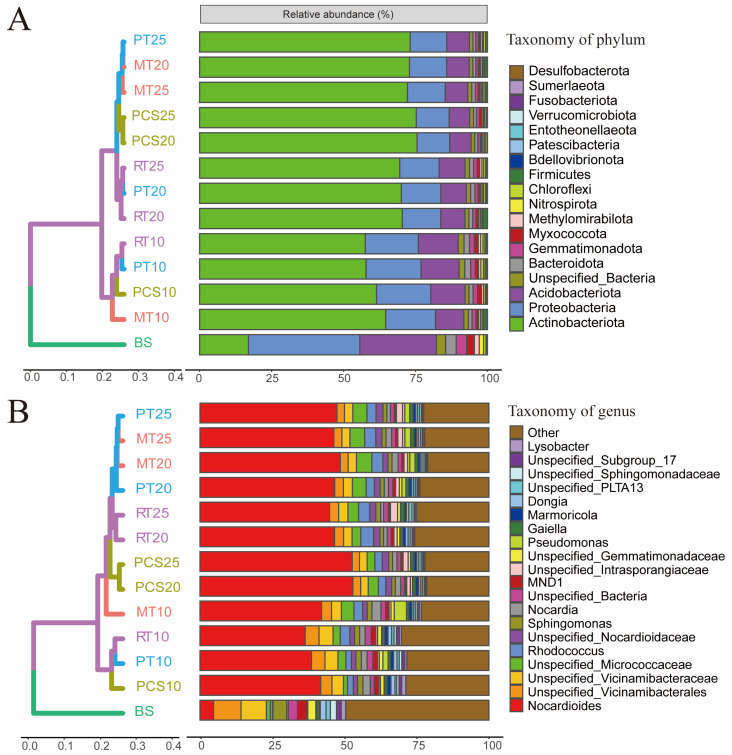
Hierarchical cluster tree and microbial composition of the BS soil and the four bioremediation groups at different sampling times based on the mean relative abundance at the phylum level (**A**) and genus level (**B**).

During bioremediation, the soil microbial community changes significantly due to the changes in soil physicochemical properties and the addition of exogenous microorganisms. In the BS group, Acidobacteriota, Actinobacteriota, and Proteobacteria were the dominant phyla, with relative abundances of 26.7 ± 1.3%, 17.0 ± 3.0%, and 38.7 ± 1.5%, respectively (Figure 3A). The relative abundance of Actinobacteriota in the restored soil was markedly increased, ranging from 57.6 ± 5.3% (RT10) to 75.6 ± 8.1% (PCS20), which is similar to the results of [54]. Actinobacteriota is a common phylum that plays an important role in the mineralization of organic pollutants [55,56]. The abundance of Proteobacteria and Acidobacteriota in the soil decreased after remediation, with the abundance of Proteobacteria being between 11.3 ± 4.0% (PCS20) and 19.1 ± 1.8% (PT10), while that of Acidobacteriota was between 7.1 ± 2.0% (PCS25) and 13.2 ± 1.2% (PT10). Based on the diversity of the microbial phylum composition in different samples, the BS group samples on day 10 and those on days 20 and 25 formed three branches (Figure 3A), indicating that the bioremediation time had a significant influence on the microbial phylum-level community structure.

At the genus level, the abundance of *Nocardioides* in the restored soil was between 36.4 ± 4.5% (RT10) and 53.0 ± 9.2% (PCS20), which was dramatically higher than that in the BS group (4.7 ± 2.5%) (Figure 3B). Many *Nocardioides* spp. can degrade crude oil [57,58]. Several studies have indicated that *Nocardioides* is often found in high abundance in petroleum-contaminated soils and is considered a promising candidate for petroleum hydrocarbon degradation [59]. However, our experimental results demonstrated that although the abundance of *Nocardioides* increased in all treatment groups, the petroleum degradation efficiency of the PCS group was lower than that of the RT and MT groups (Table 1). This suggested that the elevated presence of *Nocardioides* contributed minimally to petroleum degradation in this study. Therefore, the ecological function of *Nocardioides* in the bioremediation of petroleum-contaminated soil still requires further research. The relative abundance of the genera Unspecified_Vicinamibacterales and Unspecified_Vicinamibacteraceae decreased after remediation.

**Figure 4:**
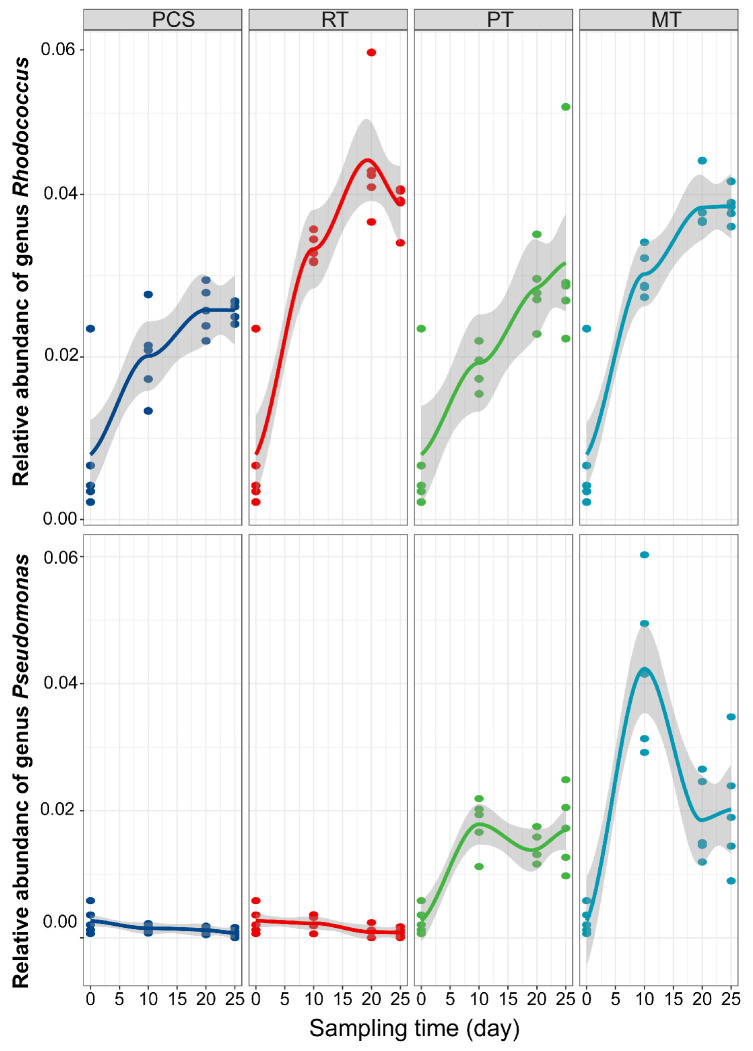
Relative abundance of genus *Rhodococcus* and *Pseudomonas* of the four bioremediation groups at different sampling times. Curve fitting was performed using the Loess method.

To investigate the effects of *Pseudomonas* sp. P35 and *Rhodococcus* sp. OS62-1 on the relative abundance of the genera *Rhodococcus* and *Pseudomonas* in soil, we analyzed the relative abundance of the genera *Rhodococcus* and *Pseudomonas* in samples treated at different times based on the ASV annotation (Figure 4). When only the KNO_3_ solution was added (PCS group), the abundance of *Rhodococcus* in the soil showed an upward trend, while the abundance of *Pseudomonas* basically did not change and had a low relative abundance. Many *Rhodococcus* spp. can degrade organic pollutants such as crude oil; thus, the relative abundance of *Rhodococcus* typically increased in crude oil-polluted soils [54,60]. Following the addition of *Rhodococcus* sp. OS62-1 (RT group), the relative abundance of genus *Rhodococcus* increased from day 5 to 20 and then decreased on day 25. A decrease in *Rhodococcus* indicates the eco-safety of *Rhodococcus* sp. OS62-1, which naturally becomes extinct when bioremediation is complete [61]. Following the addition of *Rhodococcus* sp. OS62-1 and *Pseudomonas* sp. P35 (MT group), the relative abundance of *Rhodococcus* maintained a stable state in the later stages without a downward trend. In the PT group, the relative abundance of *Pseudomonas* increased significantly during the first 10 days, and then fluctuated. In the MT group, the relative abundance of *Pseudomonas* was highest on the 10th day and was clearly higher than that in the PT group. On days 20 and 25, the relative abundance of *Pseudomonas* was clearly reduced, and its relative abundance was similar to that in the PT group (Figure 4).

**Figure 5:**
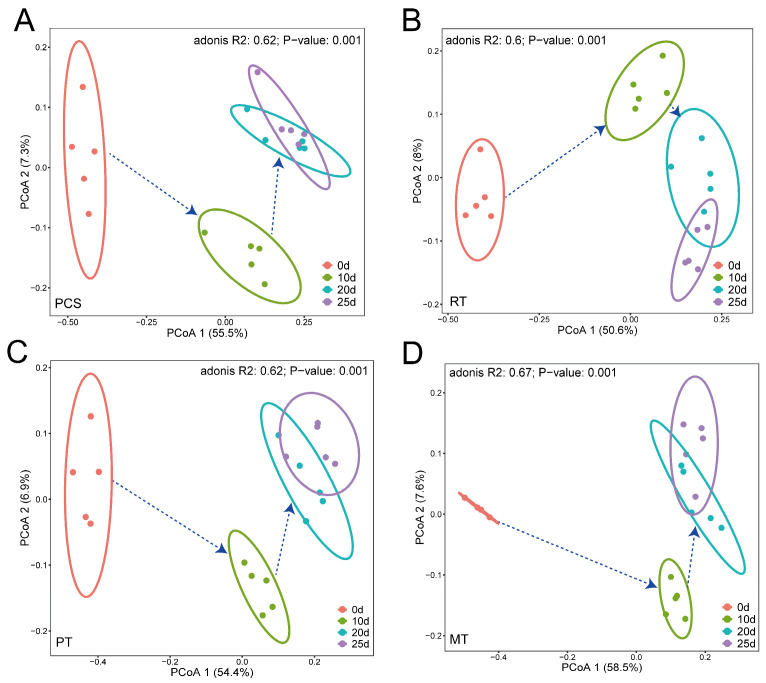
Principal coordinates analysis (PCoA) based on Bray–Curtis distance metrics of each treatment: (**A**) PCS, (**B**) RT, (**C**) PT, and (**D**) MT groups. R- and *p*-values are listed based on the results of the Adonis test. The dotted arrows indicate the time sequence.

Based on the principal coordinate analysis (PCoA), a clear shift in the microbial community structure was observed in the four different treatments (Figure 5). Furthermore, the permutational multivariate analysis of variance (ADONIS) test based on the Bray–Curtis distance measures showed that the microbial community structures were significantly (*p* = 0.001) different at each time point in all four treatments, indicating that the microbial community structure shifted during the remediation process. However, the samples collected on days 20 and 25 showed a certain overlap, indicating that the microbial community structure of these samples possessed some similarities, which might have been caused by the shorter time interval.

**Figure 6:**
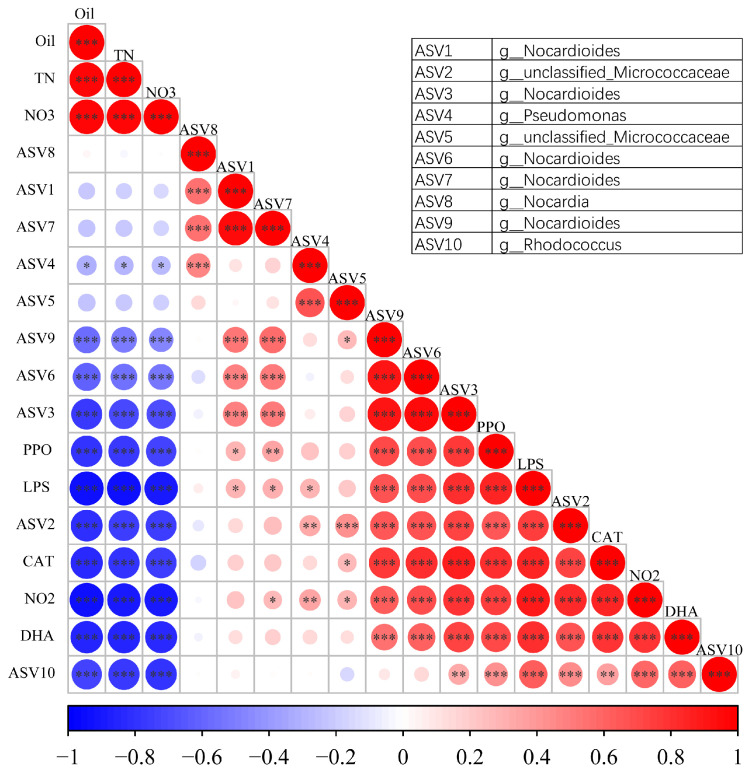
Pearson’s correlation analysis among the top 10 amplicon sequence variants (ASVs; bacterial species), oil content, and enzymatic activities. The ASVs and the corresponding species are listed at top right corner. Asterisks *, **, and *** indicate significant correlations at *p* < 0.05, *p* < 0.01, and *p* < 0.001, respectively. The red and blue colors indicate positive and negative correlations, respectively.

Correlation analyses of the top 10 dominant bacterial species, residual crude oil content (Oil), total nitrogen (TN), nitrate content (NO_3_), nitrite content (NO_2_), and enzymatic activities were performed. The results showed that *Rhodococcus* sp. (ASV10) was significantly negatively correlated with Oil, TN, and NO_3_, and positively correlated with ASV2, ASV3, PPO, LPS, CAT, DHA, and NO_2_ (Figure 6), indicating that *Rhodococcus* sp. OS62-1 enhances the growth and metabolism of some indigenous microorganisms, promotes biodegradation-related soil enzyme activity, and enhances crude oil degradation. *Pseudomonas* sp. (ASV4) was also significantly negatively correlated with Oil, TN, and NO_3_. However, *Pseudomonas* sp. (ASV4) was positively correlated with ASV2, ASV5, ASV8, LPS, and NO_2_, which may explain why oil degradation by *Pseudomonas* sp. P35 was weaker than that of *Rhodococcus* sp. OS62-1. Combined with the relative abundance of *Rhodococcus* (Figure 4), the correlation analyses showed that *Rhodococcus* could not only remediate oil pollution by itself, but also improve oil remediation by promoting the metabolic activity of indigenous microorganisms. ASV1 (assigned to *Nocardioides* sp.) showed a significant positive correlation with other *Nocardioides* species (ASV3, ASV6, ASV7, ASV9) and with ASV8 (assigned to *Nocardia* sp.), suggesting potential cooperative interactions or functional associations among these strains. However, the relationships among the *Rhodococcus*, *Pseudomonas*, and *Nocardioides* still require further in-depth research to be fully elucidated. Overall, these results revealed that the functions of the microbial community were enhanced by the bioremediation treatments.

#### 3.3.3. Microbial Networks Analysis

**Figure 7:**
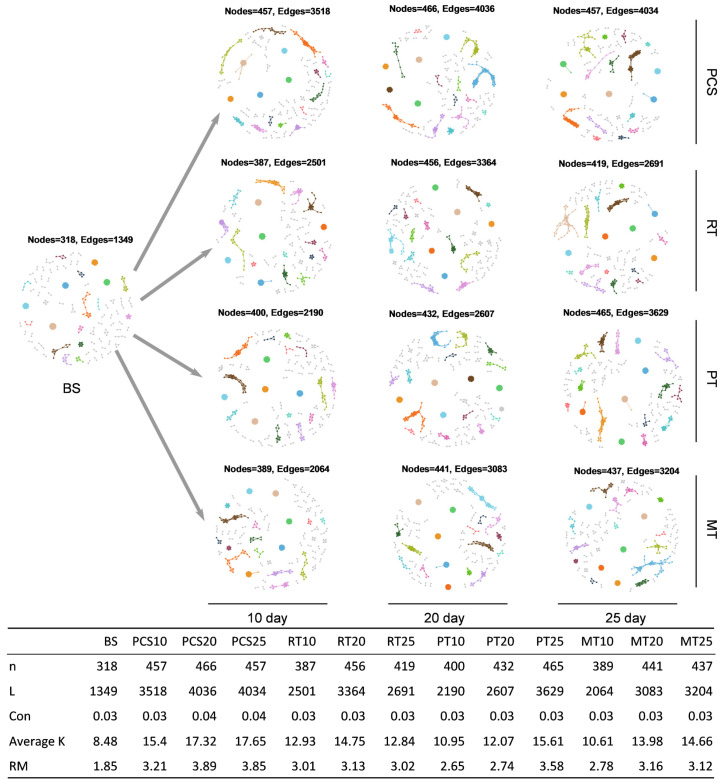
Succession of soil microbial networks during bioremediation process (**top**), and the network topology parameters of each sample (**below**), including total number of nodes (n), total number of links (L), the proportion of realized links in all possible links (Con), average links per node (average K), and relative modularity (RM).

To determine how different treatments affected the microbial network complexity, the MENs were constructed at different time points (Figure 7). Network size (total number of nodes, *n*) increased under all four bioremediation treatments compared with the BS group. Furthermore, average connectivity (average links per node and average K), network connectivity (total number of links, L), and relative modularity (RM) showed that the MENs were more complex and the interactions between microorganisms were stronger after bioremediation [28]. However, the connectance (the proportion of realized links to all possible links, Con) was maintained during the bioremediation process, indicating that the robustness of the MENs in the different treatments was similar [62]. At each sampling time point in the four different treatments, the average K of the PCS group was the highest (Figure 7), indicating that the addition of nutrients could stimulate microorganisms in the soil to cooperate closely to metabolize oil [62]. After the addition of exogenous microorganisms, the average K value decreased, indicating that the addition of exogenous microorganisms reduced the interaction between indigenous soil microorganisms.

## 4. Conclusions

This study reports the enhanced bioremediation of petroleum-contaminated soils by nitrogen source, *Rhodococcus* sp. OS62-1, *Pseudomonas* sp. 35, and the nitrogen supplement combined with the consortium achieved the highest bioremediation efficiency because of the enhanced nitrate use and the soil enzymes. Moreover, the weak petroleum-degrading bacterium *Pseudomonas* sp. 35 can act synergistically with *Rhodococcus* sp. OS62-1, thereby improving the overall bioremediation performance. This combined remediation approach increases microbial metabolic activities, enriches indigenous degrading populations, modifies community structures, and enhances the complexity of the microbial network. The results of this study contribute to the establishment of an efficient bioremediation strategy for petroleum-contaminated soils. However, this study has a limitation in that it was conducted under controlled laboratory conditions, which may not fully reflect the actual remediation effects in complex natural soil environments with variable temperature, moisture, and indigenous microbial composition. Future research should be conducted under a natural environment and could employ an integrated approach combining metagenomics, metatranscriptomics, and metabolomics to enable a more in-depth and comprehensive investigation into the cooperative mechanisms of microbial consortia.

## Figures and Tables

**Table 1:** Soil physicochemical properties under different treatments.

Treatments	Residual Oil Content (mg)	TN (mg/kg)	${\mathrm{NO}}_{3}^{-}$-N (mg/kg)	${\mathrm{NO}}_{2}^{-}$-N (mg/kg)
BS	5345.02 ± 162.79 a	634.69 ± 28.20 a	553.08 ± 44.66 a	7.69 ± 4.32 f
PCS10	4991.36 ± 114.77 b	592.89 ± 18.84 ab	526.91 ± 25.09 ab	10.44 ± 5.81 f
PCS20	4371.80 ± 113.33 cd	529.13 ± 12.98 c	469.21 ± 37.98 bc	24.58 ± 3.20 e
PCS25	4214.33 ± 122.71 d	474.79 ± 17.32 d	457.52 ± 30.09 c	25.53 ± 3.87 e
PT10	4603.74 ± 119.04 c	556.19 ± 29.26 bc	473.26 ± 17.91 bc	12.98 ± 2.39 f
PT20	3741.81 ± 141.54 e	475.69 ± 19.61 d	375.22 ± 24.18 d	37.62 ± 4.83 d
PT25	3508.78 ± 192.59 e	435.55 ± 20.23 de	352.82 ± 24.99 d	43.36 ± 5.62 cd
RT10	4224.30 ± 158.46 d	454.11 ± 21.70 d	371.01 ± 13.46 d	16.48 ± 2.77 ef
RT20	2827.32 ± 167.51 f	296.66 ± 17.06 f	230.11 ± 22.08 e	46.11 ± 3.60 bcd
RT25	2468.79 ± 148.22 g	264.97 ± 25.21 fg	173.39 ± 18.90 ef	49.80 ± 4.25 abc
MT10	3638.95 ± 150.65 e	387.33 ± 26.98 e	341.47 ± 25.56 d	39.87 ± 5.20 cd
MT20	2271.87 ± 137.63 gh	229.72 ± 14.50 g	152.61 ± 32.95 f	54.11 ± 2.15 ab
MT25	1973.17 ± 169.29 h	158.66 ± 15.17 h	115.78 ± 15.71 f	58.14 ± 6.62 a

Values are presented as the mean ± standard deviation (n = 5). Different lowercase letters within a column for the same factor indicate significant differences (*p* < 0.05) based on a one-way ANOVA, followed by a Tukey’s test. TN, total nitrogen; ${\mathrm{NO}}_{3}^{-}$-N, soil nitrate nitrogen; ${\mathrm{NO}}_{2}^{-}$-N, soil nitrite nitrogen.

**Table 2:** Alpha-diversity indices of microbial communities in different treatments.

Sample ID	Chao1	Shannon	Simpson
BS	523.0 ± 29.9 a	8.5 ± 0.1 a	0.99 ± 0.02 a
PCS10	450.2 ± 25.1 b	6.4 ± 0.4 bc	0.89 ± 0.03 bc
PCS20	424.4 ± 55.0 b	5.9 ± 0.7 c	0.87 ± 0.05 c
PCS25	422.8 ± 46.6 b	6.1 ± 0.5 bc	0.90 ± 0.03 bc
RT10	471.0 ± 50.7 ab	6.8 ± 0.3 b	0.92 ± 0.02 b
RT20	441.8 ± 32.0 b	6.3 ± 0.3 bc	0.90 ± 0.02 bc
RT25	457.2 ± 16.8 ab	6.7 ± 0.2 b	0.94 ± 0.01 b
PT10	482.0 ± 29.7 ab	6.7 ± 0.2 b	0.91 ± 0.01 b
PT20	460.2 ± 52.5 ab	6.3 ± 0.5 bc	0.90 ± 0.03 bc
PT25	426.8 ± 34.6 b	6.4 ± 0.4 bc	0.92 ± 0.02 bc
MT10	463.2 ± 16.9 ab	6.3 ± 0.2 bc	0.90 ± 0.02 bc
MT20	427.6 ± 41.4 b	6.1 ± 0.5 bc	0.90 ± 0.04 bc
MT25	430.0 ± 13.8 b	6.5 ± 0.1 bc	0.93 ± 0.01 bc

Values are presented as the mean ± standard deviation (n = 5). Different lowercase letters within a column for the same factor indicate significant differences (*p* < 0.05) based on a one-way ANOVA, followed by a Tukey’s test.

## Data Availability

Data available in a publicly accessible repository. The data presented in this study are openly available in [SRA] at [https://www.ncbi.nlm.nih.gov/sra/?term=SRR29243594], reference number [SRR29243594–SRR29243658].
